# Philadelphia Veterinary Medical Association

**Published:** 1892-12

**Authors:** 


*The Philadelphia Veterinary Medical Society* was called to order by President S. J. J. Harger. Members present: Drs. Ridge, Maher, Bunting, Conard, Williams, Eves, Magill, Pearson and Conrow. Visitors: Drs. John Marshall, T Earl Budd and Herman Wellner.

New members elected: Francis S. Allen, M.D.,V.S., T. Earl Budd, D.V.S., Horace P. Keely, V.M.D.

Dr. Leonard Pearson read paper upon anthrax, discussed by Drs. Eves and Williams. Drs. Ridge and Conard cited cases of stoppage of œsophagus in cows.

Dr. Magill cited a case in a horse. Dr. Conard cited cases of contagious conjunctivitis and keratitis in cattle. Treatment, 2½ per cent. creolin sol. Bandage, atropia and boracic acid sol.

Dr. T. Earl Budd reported a case of dystokia in bitch due to fractured pelvis. Dr. S. J. J. Harger performed Caesarean section.

Dr. Ridge moved to suspend by-laws. Carried.

Dr. Conard offered resolutions protesting against the starting of a two-year Veterinary School at Washington, D. C., by employes of Bureau of Animal Industry. Discussed by Drs. Marshall, Ridge, Eves, Williams, Pearson, Budd and Harger. Moved by Dr. Maher to adopt as read and to send copies to Bureau of Animal Industry, Secretary of Agriculture and Secretaries of Veterinary Societies. Seconded by Dr. Eves. Carried. Adjourned to meet October 25th.

E. E. CONROW, *Secretary.*

*The Philadelphia Veterinary Medical Society* was called to order by President S. J. J. Harger, October 25th at the College of Physicians. Members present: Drs. Ridge, Schreiber, Landis, Conard, Entriken, Rogers, Zuill, Keely, Holdsworth, Tag and Conrow. Visitor, Dr. J. W. Adams.

Minutes last meeting read and approved. New member elected, J. W. Adams, V.M.D.

Communications read from Drs. Cooper Curtice, of Moravia, N. Y., and M. Stalker, of Ames, Iowa, in answer to resolutions adopted at our last meeting.

On motion communications ordered filed and copies forwarded to JOURNAL and *Review* for publication. Dr. Zuill moved that Dr. Fennimore's communication be received and his name be transferred from active to corresponding membership. Carried.

Our essayist, Dr. Eves, being absent, Dr. Conard reported his cases of contagious keratitis as having done well.

Dr. Landis cited case of abscess of rectum with chronic peritonitis and pleurisy.

Dr. Conard cited case of tumor in mesentary of small intestine as large as a Yankee bucket.

Dr. Rogers reported case he thinks contagious pleuro-pneumonia. Discussed by Drs. Zuill, Schreiber, Ridge and Landis.

Dr. Zuill censured officers for not having a paper read at this meeting and insists that all cases should be presented in writing. Adjourned until November 22nd.

A. E. CONROW, *Secretary.*

IOWA STATE BOARD OF HEALTH, AMES, IOWA, Oct. 7, 1892.

A. E. CONROW, M. D.

*Dear Sir*:—A copy of the resolutions adopted by the Philadelphia Veterinary Society has been received at my office. I have also received the announcement of the Washington Veterinary School. Your society could not have experienced more surprise and disappointment on the receipt of that document than I did. I feel that the veterinary profession of this country, and especially those who have given many of the best years of their lives in an honest endeavor to elevate the cause, had a right to expect other and better things from that source. I was of the opinion we had just reached a point in the history of veterinary matters in the United States when all the better influences would be expected in the direction of more thorough courses of study, and when the lecture fee would cease to constitute the "long felt want," that impels to the organization of a new college. I had hoped that with the recent move of the United States Veterinary Medical Association in asking for higher qualifications for membership would follow a change in the curriculum of every veterinary school in this country advertising a two years course, and yet laying claim to a respectable fellowship among veterinary colleges. But it had never occured to me to entertain a fear that those in high official positions, those whose station best fits them for giving force and effect to this movement should be the first to aid in balking it.

I am in thorough accord with your association in its unqualified protest against any system which recognizes twelve months college work as adequate to secure a college degree. I am yours very truly,

M. STALKER.

WHITE PLAINS, N. Y., Oct. 9, 1892.

DR. A. E. CONROW, Moorestown, N. J.,
Sec'y Penn., Vet. Society.

*Dear Doctor:*—I have just received a copy of resolutions regarding a veterinary school to be established at Washington, D.C., by members of Bureau of Animal Industry. I have written for a prospectus which I hope to obtain soon.

The affair is one of deep interest. The vital joints to me are: not whether a set of men shall establish a 2, 3, 4 or 5 year school, but whether public business can or will be successfully and honestly prosecuted by men who have private interests in the same line at stake; also whether it is fair to other private colleges for the Government to permit men in its employ to use its patronage for their private school in even an implied way.

We will not question the motives of the founders other than to admit that they wish to increase their income by transacting private business. This is done by a large number of Government employes without a question being raised as to its legality or propriety; we can but admit that, aside from the short term graduation clause, that the prominence of the men proposing the school borrows some of its force from the position they hold. There is another deeper objection; after a year, after two years, and as the years go by, such a school will have an alumni of two year men. These men will compete with graduates of three year colleges for positions at the disposal of the Department of Agriculture and the Bureau of Animal Industry. Now it is but natural that members of the Bureau having come into contact with the students of their college would desire to place them advantageously. This would not necessarily interfere with the relative quota furnished by different States; but if taken advantage of would interfere with the rights of other graduates from other colleges equally good, if not better.

It may be claimed that their men, knowing the wants of the Bureau, could train their men better for special work. I should say that this would be further infringement of private rights; it would also tend to narrowness of views in graduates of the proposed college and a deterioration of the profession. That the professors of a veterinary school, not a Government school, should have an immense patronage derived from the Government as officers of the most powerful Bureau of the Agricultural Department is of itself an inducement to students to attend. Add to this a short course, where other schools are preparing to raise their standard, and there is attained the combination of short time graduation with the strongest possibility of an appointment in Government service accompaning the diploma.

The mere fact that such things might be is sufficient, in my opinion, to effect, that not only should the men designing such a step look very carefully into its results and the appearance it may present to those of the profession on the outside, but the Secretary of Agriculture also should investigate the apparently harmless move which gains strength from the fact that men of high standing in the Bureau are its promulgators inevitably linking the fair name of the Department with their destinies whether the business be conducted fairly or not.

That the veterinary profession should take every interest in either promoting the enterprise as an additional educational institution for the welfare of the profession, or in opposing it as being founded against the best interests of the people and the profession, follows without saying.

It must be remembered, sir, that I write this without reference to the integrity of any of the promoters of the scheme proposed, for among the Bureau are men whom I esteem my best of friends. It is simply as an institution that I write of it, judging that its originators merely misjudged the effect that the creation of a new veterinary school under their auspices would produce upon the public.

Is there not something behind all this which is suggestive? Unless we wish to ascribe the motives of the proposed faculty as benevolent we must believe them mercenary. It is true that many Government employes living in Washington feel the stress of the normal living expenses upon their salaries and to prepare for the rainy day embark into one or another enterprises to add to their income.

It is also true that members of the Bureau are paid comparatively low as compared with other departments of service and as compared with professional men of equal attainments in other walks of civil life. It is, moreover, true that various colleges of Washington draw upon the various branches of the Government for many of their professors. Have not the promoters of this enterprise plenty of precedence for their action and an added stimulus in the fact of insufficient salaries?

Is not the remedy for the Department of Agriculture in employing its men to pay them amply for professional services and exact of them that they shall expend their strength and services in their work and above all not engage in enterprises which may involve the good name and the patronage of the department?

Further, is not the desirability of a Government school of an advanced type suggested in this movement? A school for graduates of other colleges, a school manned by professors separate from the employ of the Bureau, but which may use the employes at stated intervals in clinical work; and take advantage of the exceptional facilities for advanced work to be found at Washington.

While sympathizing with your society in the belief that our profession is injured by the establishment at this time of a two year college, you will know from the above why I do not endorse your resolutions except in the last proposition that it seems "contrary to the institution of the Government to make use of one of its bureaus to establish and maintain a private school."

It seems to me that the reasons of protest should have been brought out more fully in this line as the Secretary of Agriculture should care very little whether the school be a one or a four year school, but whether he had any right to interfere with private persons (as the members of the Bureau are outside of office) setting up any business they pleased. If the movement against it is to meet with any success it must be on the plea that the school, because of its officers being in the pay of the Government, implicates and involves the Government in some way. If this can be established *i. e.* if it can be shown that a private enterprise gains prestige and patronage from the Government with the consent of those interested only you will soon see the head of the Department involved set the necessary machinery in motion to rectify the evil complained of. Believe me sir, Very truly yours,

COOPER CURTICE, Moravia, New York.

Resolutions adopted at a regular meeting of the Philadelphia Veterinary Society, September 27, 1892.

*Whereas*, It has come to our notice that the officers and staff of the Bureau of Animal Industry connected with one of the departments of the National Government, propose establishing a Veterinary School in Washington; and

*Whereas*, All prominent veterinarians agree that it is absolutely necessary for intelligent and well prepared young men to study at least three years to fit themselves to meet the multitudinous and intricate duties of the veterinary profession, and all reputable schools of this country now require three years attendance or propose inaugurating three year courses of study during the coming session ; and

*Whereas*, All of the veterinary schools of Europe require at least three years, attendance and some of them four and five years, and the Royal College of Veterinary Surgeons of England has recently adopted a four year plan of study, and the United States Veterinary Association, at its recent meeting in Boston, (Sept. 21, 1892), declared in favor of a three year course of study and made this a necessary qualification for future membership in the Association ; thus clearly showing the position of the best element of the veterinary profession; and

*Whereas*, We have a right to expect the Bureau of Animal Industry of the Department of Agriculture, as the only department of our Government under the direction of veterinarians, to use all of its influence to elevate the veterinary profession of the land and not, by permitting its officers and staff to establish and maintain a new two year, short term veterinary school use its powers to discourage thorough Veterinary Education in the United States and neutralize the progress of the last ten years ; and

*Whereas*, It seems to us contrary to the institutions of our Government to make use of one of the Bureaus of the Government to establish and maintain a school, be it

*Resolved*, That this society recognizes the utter impossibility of imparting sufficient veterinary instruction to students in two college sessions to enable them to practice veterinary medicine and surgery intelligently, and that it regards the establishment and maintainance of schools giving such courses of instruction as a menace to the progress of Scientific Veterinary Medicine. Be it further

*Resolved*, That this society urge the officers and staff of the Bureau of Animal Industry, who are about establishing a new Veterinary College in Washington requiring but two sessions attendance upon instruction, to reconsider their action and to refrain from doing that which will tend to lower the standard of the veterinary profession, retard the progress of veterinary education and lessen the dignity and standing of graduates of American Veterinary Colleges, in this and foreign countries. Be it further

*Resolved*, That this society protests against such use of the Bureau of Animal Industry, and that the Hon. Jeremiah P. Rusk, Secretary of Agriculture, be respectfully requested to prohibit this attempt to use the Bureau as above described. Be it further

*Resolved*, That a copy of these resolutions be sent to the Secretary of Agriculture, the Chief of the Bureau of Animal Industry and to each of the Veterinary Journals. (Signed),

A. E. Conrow, M.D., V.M.D.,
Moorestown, Burlington Co., N. J.

